# Supplementary material for: Chemically Mediated Microbial “Gardening” Capacity of a Seaweed Holobiont Is Dynamic
Source: Microorganisms. 2020 Nov 30;8(12):1893. doi: 10.3390/microorganisms8121893 (PMC7760634; doi:10.3390/microorganisms8121893)
Supplement: Supplementary file 1 [file microorganisms-08-01893-s001.zip › microorganisms-954147-suppplementary/microorganisms-954147-suppplementary-original.pdf]

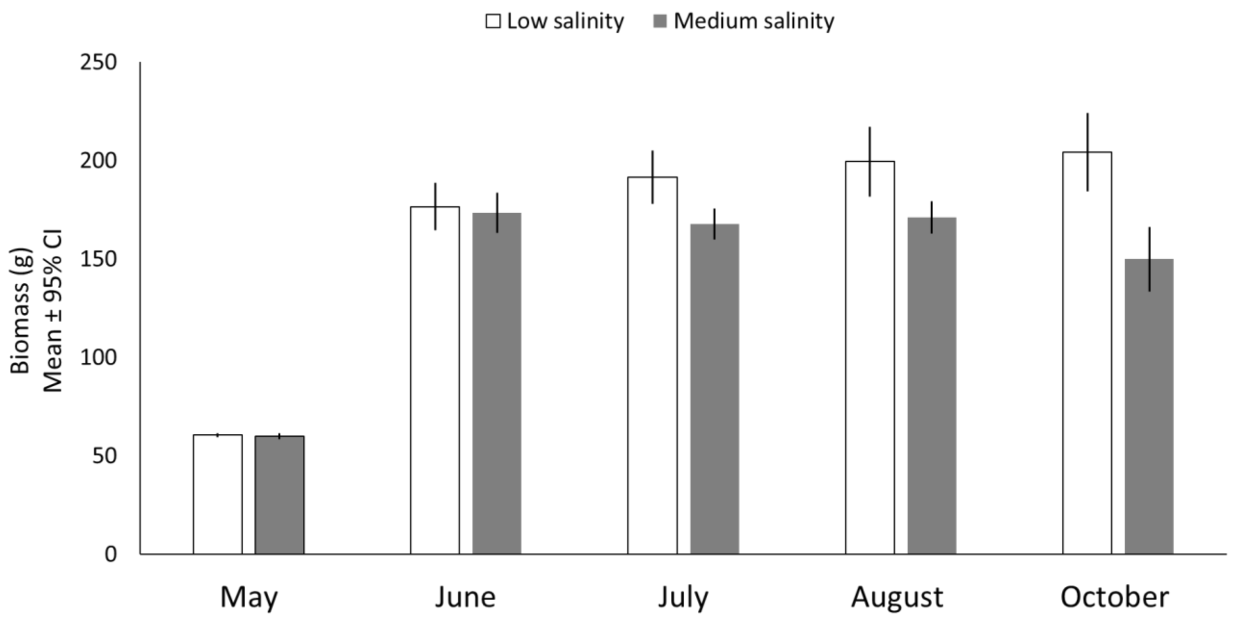

Figure S1: Wet weight of *Agarophyton* between start and end of the experiment at both salinity levels. Error bars ± CI (n=5)
